# Providers’ perceptions of disrespect and abuse during childbirth: a mixed-methods study in Kenya

**DOI:** 10.1093/heapol/czaa009

**Published:** 2020-03-10

**Authors:** Patience A Afulani, Ann Marie Kelly, Laura Buback, Joseph Asunka, Leah Kirumbi, Audrey Lyndon

**Affiliations:** c1 Department of Epidemiology and Biostatistics, University of California, San Francisco (UCSF) School of Medicine, 550 16th St, San Francisco, CA 94158, USA; c2 UCSF Institute for Global Health Sciences, 550 16th St, San Francisco, CA 94158, USA; c3 Sidney Kimmel Medical College, 1025 Walnut St, Philadelphia, PA 19107, USA; c4 The William and Flora Hewlett Foundation, 2121 Sand Hill Road, Menlo Park, CA 94025, USA; c5 Kenya Medical Research Institute, Mbagathi Rd, Nairobi, Kenya; c6 New York University Rory Meyers College of Nursing, 433 First Avenue, New York, NY 10010, USA

**Keywords:** Disrespect and abuse, mistreatment, respectful maternity care, person-centred maternity care, quality of care, maternity providers, Kenya

## Abstract

Disrespect and abuse during childbirth are violations of women’s human rights and an indicator of poor-quality care. Disrespect and abuse during childbirth are widespread, yet data on providers’ perspectives on the topic are limited. We examined providers’ perspectives on the frequency and drivers of disrespect and abuse during facility-based childbirth in a rural county in Kenya. We used data from a mixed-methods study in a rural county in Western Kenya with 49 maternity providers (32 clinical and 17 non-clinical) in 2016. Providers were asked structured questions on disrespect and abuse, followed by open-ended questions on why certain behaviours were exhibited (or not). Most providers reported that women were often treated with dignity and respect. However, 53% of providers reported ever observing other providers verbally abuse women and 45% reported doing so themselves. Observation of physical abuse was reported by 37% of providers while 35% reported doing so themselves. Drivers of disrespect and abuse included perceptions of women being difficult, stress and burnout, facility culture and lack of accountability, poor facility infrastructure and lack of medicines and supplies, and provider attitudes. Provider bias, training and women’s empowerment influenced how different women were treated. We conclude that disrespect and abuse are driven by difficult situations in a health system coupled with a facilitating sociocultural environment. Providers resorted to disrespect and abuse as a means of gaining compliance when they were stressed and feeling helpless. Interventions to address disrespect and abuse need to tackle the multiplicity of contributing factors. These should include empowering providers to deal with difficult situations, develop positive coping mechanisms for stress and address their biases. We also need to change the culture in facilities and strengthen the health systems to address the system-level stressors.

**Key Messages**

Many maternity providers have witnessed or engaged in disrespect and abuse of women during childbirth.
Disrespect and abuse are driven by difficult situations in a health system and sociocultural environment that facilitates it. Providers resorted to disrespect and abuse as a means of gaining compliance when they were stressed and feeling helpless.
Provider implicit biases influence the patient–provider interaction, leading to differential treatment of women during childbirth.
Interventions to address disrespect and abuse need to tackle the multiplicity of factors that drive and facilitate it. This should include empowering providers to deal with difficult situations as well as changing the culture in facilities and strengthening the health system to address the system-level stressors.

## Introduction

Improving quality of care is a global priority for maternal and neonatal health (Koblinsky *et al.*, 2016). The World Health Organization’s (WHO) vision for quality of maternal and newborn health highlights three domains under the experience dimension of quality of care: dignity and respect, communication and emotional support (Tunçalp *et al.*, 2015). Dignity and respect emphasize care that maintains women’s dignity, ensures privacy and confidentiality and provides freedom from mistreatment such as physical and verbal abuse and discrimination (WHO, 2018).

Disrespect and abuse during childbirth are a violation of women’s human rights, and an indicator of poor-quality care (WHO, 2015). Yet, many women experience disrespect and abuse during childbirth (Bohren *et al.*, 2015). Given that the target of disrespect and abuse is women, research has largely (and understandably so) focused on the perspectives of women, with relatively little attention to provider perspectives. The few studies with providers find that most providers have witnessed some form of disrespect and abuse in their facilities in both low- and high-resource settings (Asefa *et al.*, 2018; Morton *et al.*, 2018). A better understanding of provider attitudes and behaviour is, however, essential for identifying the drivers of disrespect and abuse and informing interventions to address such behaviours.

The potential drivers of disrespect and abuse operate at various levels. At the individual level, providers state that disrespect and abuse are unintended and are justified as necessary to help women in the birthing process (Bohren *et al.*, 2016; Burrowes *et al.*, 2017; Warren *et al.*, 2017). Others blame women’s disobedience and lack of co-operation (Bohren *et al.*, 2016; Rominski *et al.*, 2017). In a study in Nigeria on acceptability of mistreatment during childbirth, Bohren *et al.* (2016) found that while some respondents viewed scenarios such as slapping, verbal abuse and physical restraint as abuse, others thought these were acceptable means of gaining compliance to ensure a good outcome. The role of power asymmetry, institutional structures, social and economic inequality, social and gender norms, and normalization in disrespect and abuse have also been described (Jewkes *et al.*, 1998; Freedman *et al.*, 2014; Jewkes and Penn-Kekana, 2015; Sen *et al.*, 2018). Much is, however, yet to be learned about the drivers of disrespect and abuse and listening to service providers is essential to extending this knowledge (WHO *et al.*, 2016).

In this article, we seek to examine the extent and drivers of disrespect and abuse during facility-based childbirth from the perspectives of maternity care providers in a rural county in Kenya. The primary research questions were the following: (1) what are providers’ perceptions of the extent of disrespect and abuse in their facilities? And (2) what are the drivers of disrespect and abuse?

## Methods

The data are from a larger mixed-methods project in a rural county in western Kenya to understand community perceptions of quality of maternity care (Afulani *et al.*, 2017b; Afulani *et al.*, 2018a,b). The setting, methods and user perspectives are described in detail elsewhere (Afulani *et al.*, 2017b; Afulani *et al.*, 2018a,b). The data presented here are from interviews with 49 maternity providers from 18 facilities across all the eight sub-counties. Providers were purposefully selected and interviewed in October and November 2016. The facilities were selected for an intrapartum quality improvement project based on their higher volume of births. Two to four providers were selected from each facility to include different cadres of staff. We used a convergent mixed-methods design to address both research questions (Creswell, 2014). Two research assistants conducted the interviews using a guide containing both structured and open-ended questions. Interviews were conducted in English, Swahili or Luo—in private spaces in each health facility—and lasted about an hour. The structured responses were directly entered into REDCap (Harris *et al.*, 2009). Interviews were audio-recorded and transcribed (with simultaneous translation where necessary). Ethical approval was obtained from the authors’ institutions and all participants provided written informed consent.

We operationalized dignity and respect with questions adapted from the person-centred maternity care scale (Afulani *et al.*, 2017a), which has three subscales: dignity and respect, communication and autonomy, and supportive care. These subscales capture the three domains of experience of care in the WHO vision for maternal and newborn health (Tunçalp *et al.*, 2015). In this article, we conduct a distinct analysis of provider responses related to dignity and respect, which include questions on respectfulness, friendliness, verbal and physical abuse, privacy and confidentiality, with additional questions on discrimination and detainment (Table 2).

**Table 2:** Provider perceptions of extent of disrespect and abuse (*N* = 49)

Construct	Question	No, never, *N* (%)	Yes, a few times, *N* (%)	Yes, most of the time, *N* (%)	Yes, all the time, *N* (%)
Respect	Do the doctors, nurses or other staff at the facility treat women with respect?	0 (0)	4 (8.2)	26 (53.1)	19 (38.8)
Friendliness	Do the doctors, nurses and other staff at the facility treat women in a friendly manner?	0 (0)	3 (6.1)	35 (71.4)	11 (22.4)
Verbal abuse	Do the doctors, nurses or other health providers shout at, scold, insult, threaten or talk to women rudely?	20 (40.8)	19 (38.8)	9 (18.4)	1 (2.0)
	In your experience at this facility, have you seen this happen?	17 (34.7)	26 (53.1)	4 (12.2)	0 (0)
	In your experience at this facility, have you ever done this?	23 (46.9)	22 (44.9)	4 (8.2)	0 (0)
Physical abuse	Are women treated roughly like pushed, beaten, slapped, pinched, physically restrained or gagged when they are delivering in the health facility?	35 (71.4)	13 (26.5)	1 (2.0)	0 (0)
	In your experience at this facility, have you seen this happen?	31 (63.3)	18 (36.7)	0 (0)	0 (0)
	In your experience at this facility, have you ever done this?	32 (65.3)	17 (34.7)	0 (0)	0 (0)
Privacy and confidentiality	During examinations in the labour room, are women covered up with a cloth or blanket or screened with a curtain so that they do not feel exposed?	1 (2.0)	11 (22.4)	13(26.5)	24 (49)
	Do you think women need privacy during their time in the labour ward?	0 (0)	2 (4.2)	13 (27.1)	33 (68.8)
	When women are speaking to the doctors, nurses or other staff at the facility, do you think other people not involved in their care can hear what they are discussing?	20 (40.8)	14 (28.6)	12 (24.5)	3 (6.1)
	Do you feel like women’s health information is kept confidential at this facility?	1 (2.0)	2 (4.1)	23 (46.9)	22 (44.9)
Differential treatment	Will you say women are sometimes treated differently because of their personal attributes, like their age, marital status, number of children, education, wealth, their connections with the facility or things like that?	41 (85.4)	2 (4.2)	5 (10.4)	0 (0)
	Do you think you sometimes treat women differently based on some attributes like their education, wealth, age, marital status, their connections with the facility or things like that without being aware of it?	27(61.4)	14(31.8)	3 (6.8)	0(0)
Detention in facility	Are women forced to stay at the health facility against their will because they cannot pay?	41 (83.7)	5 (10.2)	3 (6.1)	0 (0)

### Data analysis

We characterized the sample and structured question responses using descriptive statistics and conducted thematic analysis for the qualitative data (Braun and Clarke, 2006). The first three authors (and another research assistant initially) coded the transcripts and wrote analytic and reflexive memos to capture their reactions to the data and emerging ideas. We then analysed the codes and coded text and reviewed our memos to generate categories and identify themes and selected representative quotes to illustrate the range of voices in each theme. We considered both the semantic (surface) and latent (underlying) meaning of the text and focused on salience rather than frequency in the qualitative analysis. Quantitative data were analysed in STATA 15 (StataCorp, 2017) and qualitative data in ATLAS.ti (2016).

## Results

The respondents included 32 clinical providers (7 clinical officers/doctors and 25 nurses/midwives) and 17 non-clinical staff (cleaners, cooks and ward aids). Thirty providers worked in government hospitals, 13 in government health centres and 6 in mission/private facilities (selected demographics shown in Table 1 and by provider and facility type in Supplementary Appendix 1). The quantitative results assess providers’ perceptions of the extent of disrespect and abuse and the qualitative data assess their perceptions of the drivers of disrespect and abuse.

**Table 1:** Distribution of provider characteristics (*N* = 49)

	No.	%
Facility type		
Government hospital	30	61.2
Government health centre	13	26.5
Mission hospital	6	12.2
Position		
Clinical officer/doctor	7	14.3
Nurse/midwife	25	51.0
Support staff	17	34.6
Female	35	71.4
Age (year)		
<30	9	18.4
30–39	21	42.9
>39	19	38.8
Married	39	83.0
Years as provider		
>6	18	36.7
6–10	13	26.5
>10	18	36.7
Works >5 days a week	11	22.9
Works >8 h per day	23	47.9
From county	29	59.2
<10 years in county	14	28.6

### Extent of dignified and respectful care

In general, providers reported that women were treated with respect most or all the time: 53% said women were treated with respect most of the time and 39% said all the time (Table 2). However, they acknowledged that women were sometimes verbally or physically abused by providers and some admitted that they had ever verbally or physically abused a woman themselves: 65% reported seeing verbal abuse and 53% had personally verbally abused a woman. In addition, 37% reported seeing physical abuse and 35% had personally physically abused a woman.

About half reported that women were always covered or screened off during examinations. But 41% reported women could never talk to providers without other people overhearing. Most providers did not acknowledge discrimination based on personal attributes, though about 40% acknowledged it when phrased as occurring ‘without being aware of it’. About 16% reported that women were sometimes detained because they were unable to pay for services (mostly in the private facilities—Supplementary Appendix 2).

### Drivers of disrespect and abuse

We identified five themes from providers’ reasons for disrespect and abuse: *difficult and unco-operative women*, *environmental and situational factors*, *provider attitudes*, *provider bias* and *provider training and women’s empowerment.* These themes interact with each other in several ways.

#### Perceived difficult and unco-operative women

The most common reason providers gave for verbal and physical abuse (shouting at, threatening, pinching or slapping women) was that they ‘had to do it’ to save the baby when the woman was unco-operative or difficult. Women who did not follow their instructions (e.g. to expose their perineum, to push or not to push), refused examinations or aspects of care, screamed too much, wanted to deliver on the floor, were impatient or insisted on being seen ahead of others, or were disrespectful to providers were described as difficult. Women having their first birth were described as more likely to be difficult.

*It [verbal abuse] happens on a few cases where the mother is refusing to cooperate during delivery…We are not doing this to harm the mother but to save the life of the baby* (NC3).

There was a sense that ‘difficult’ women prevented providers from executing their role, which is to deliver live babies, and it was necessary to do whatever it took to save the baby. Providers seemed overwhelmed when they felt the baby might die because of woman’s lack of co-operation and they reacted by being verbally or physically abusive. Abuse was thereby framed as driven by good intentions for the mother.

*Sometimes they refuse to open their privacy [expose perineum] in second stage and the child is getting to distress and you are seeing the head…so sometimes it forces you to tell her that you are killing the child. Sometimes you may be soft and the child dies. Giving birth is very hard, bringing out a child especially to a primi is not a joke and you make sure the child is coming out alive. Sometimes these ladies scream at the top of their voices [and] you can’t talk to such a person. Instead you shout…sometimes you can pinch…* (C38).

Providers’ attitudes towards ‘difficult’ women appeared to be both an expression of lack of control and an exercise of power. Many providers reported that they were ‘forced’, which suggests a level of helplessness or a perceived lack of agency in their actions. At the same time, responses such as she ‘must co-operate’ and their desire for women to always follow their instructions reflected their perceptions of being in charge and women’s perceived lack of agency. Verbal and physical abuse thus appeared to be both inherent and reactive behaviours to maintain provider control. These were considered acceptable behaviours to gain compliance—and providers had the power to use them. To some, hitting a woman was a last resort to secure compliance and they rationalized it as being in the mother’s interests.

*…. When the mother is uncooperative especially during second stage, yes like in my instance why I was forced to pinch, we had a tight cord around the neck, this mother was a para six [six prior births] and after the head had crowned you are telling her not to push so that you can clamp the cord and cut but she insisted on pushing so I had to pinch her kidogo [a little]* (C4).

Perceiving women as difficult or unco-operative evoked feelings of anger, hostility, irritation, unhappiness and fear from providers. Providers were also angered by behaviours such as delays in seeking care and using traditional providers and treatments. Providers described anger leading to verbal abuse including threats of giving women episiotomies. In such cases, providers blamed their abusive behaviours on the woman.

*… sometimes I get annoyed because here is a case where somebody has labored four days at home, she has been taken to the Traditional Birth Attendants, she has taken local herbs, something like that we get annoyed, that one is true we get annoyed…we do shout to them [and] we even threaten them that we will do episiotomy so that they can deliver… sometimes after delivering they will say ‘when I went there I was mistreated, I was done that’, but it is their own making that is why we do* (C17).

Some behaviours classified as verbal and physical abuse were considered more acceptable than others. For example, one provider said, ‘you are forced to raise your voice at her, but you are not scolding her’. In addition, threatening a mother that she will lose her baby was thought by some as explaining to the mother to get her co-operation. Others also said their firmness is sometimes misinterpreted as insulting the woman. Some providers believed that a soft tone in a dire situation might be interpreted to mean the situation is not serious, so they used their facial expression and tone of voice to show the gravity of the situation. Others felt pinching or forcefully holding down a woman was acceptable to save the baby, but slapping was not. Non-clinical providers endorsed similar beliefs and behaviours.

*Allow me to be honest, I have…pinched but not slapping. I am not brutal…. sometimes we are forced to pinch but not push or slap* (C4).

Some providers described reacting to difficult or unco-operative behaviour unconsciously. Some providers also acknowledged that women were sometimes unco-operative because of pain, and verbal and physical abuse added to their pain. Others noted that explaining to the woman what they were doing might help gain co-operation, but that when the woman was still not co-operative, they used whatever means it took to save the baby.

*When the patient is not cooperative like the cases I have told you, someone is in second stage and she is still closing up her legs, you will find yourself shouting at her without knowing. Somebody is having contraction in second stage and she jumps from delivery bed to the floor because they want to deliver on the floor, so you will be forced to shout at them even unknowingly* (C11).

#### Environmental and situational factors

This theme captures references to facility and health system-related factors that contribute to disrespect and abuse and has the following three sub-themes: *Stressful work conditions and burnout*; *facility culture and accountability*; and *poor infrastructure and lack of supplies and medications.*

##### Stressful work conditions and burnout

Providers directly and indirectly cited stress and burnout as reasons for disrespect and abuse. While some providers used the terms stress and burnout, others talked about tiredness and exhaustion. Providers associated stress with forgetfulness, impatience, irritability and anger (‘feeling worked up’).

*…it comes out with the work burn out, with the stress, and sometimes you become irritable* (C4).

*Staff shortage is a big issue; you can find yourself on night duty at the same time you are covering day time, and so you can’t get good services that you want to give a client because you are exhausted* (C2).

Factors contributing to stress and burnout included high workload due to staff shortage, lack of essential supplies and medications, women presenting in labour without the recommended items, disrespect from women, families and other staff, language barriers, women not co-operating, and fear of maternal or newborn death. Women were often perceived as difficult during stressful situations (e.g. delayed second stage or a cord around the baby’s neck). In addition, providers described situations where they or other providers projected their non-work-related stress onto women.

*Maybe you call the doctor this is an emergency, and she/he has come with some stress from wherever she/he comes from, when they come, they start to pour the anger on the patient by shouting* (C10).

*Whenever there is burnout then you just find yourself not giving the clients the best that you should, you just find yourself treating the patients as if they are the cause of the burnout* (C11).

##### Facility culture and accountability

The role of facility culture was expressed across themes yet warrants specific consideration. Providers seemed more likely to engage in behaviours they felt were acceptable in their facility. Unacceptable behaviours might be punished if someone was willing to stand up against them. Unfortunately, punishment was sometimes just a transfer to another facility. Some providers thought disrespect and abuse were more likely at night when providers were often alone and unlikely to be held accountable for their behaviour.

*There is one nurse who was mistreating patients and I had to write a letter to the [Ministry of Health] and he had to be transferred…He was taking money from patients, beating [women at] the time of pushing….this one can’t happen [here]…[providers] know if they do so they have to go to another place* (C25).

##### Poor infrastructure and lack of supplies and medications

Providers noted that it was sometimes difficult to maintain women’s privacy and confidentiality because of the open nature of the labour wards, which were often too small for the number of women in labour. This was compounded by lack of privacy screens.

*…we have to treat them as individual and give them their privacy. But also because of the space that is available and we have to help all these clients, it forces you to mix them, and also as you speak to this one, the other one will also have to hear what you are saying to this one* (C5).

In addition, providers reported that facilities lacked basic supplies and medicines, and women had to bring their own supplies (cotton wool, sanitary pads, sheets, detergents, etc.) and buy medicines. Because of this, women who did not bring their own sheets were sometimes left uncovered and some providers got angry and verbally abusive towards women for not bringing the required supplies. Community expectations for ‘free maternity care’ under the country’s maternal health policy were also reported to exacerbate tensions between providers and women/families who contested having to bring supplies or buy medicines.

*Some come without anything and they are in their second stage that you can’t send them to go back and bring even a cloth… So that is when you can get so angry with a mother because you are asking her, ‘where are the babies’ clothes,’ and she is like I left at it home…sometimes you are forced to remove your scarf and wrap the baby with because the linens are also so few* (C38).

*…they [expect that] everything is free. When you tell them to go and buy, they feel bad and sometimes they abuse us. They think that these things are here, and we are not giving them…* (C3).

#### Provider attitudes

Compared with blaming disrespect and abuse on women’s behaviour and environmental factors, relatively fewer providers admitted to the role of the provider in these behaviours. However, a few providers acknowledged that disrespect and abuse of women were sometimes due to provider attitudes and temperament. For example, some providers were said to be rude or arrogant. Provider attitudes were attributed to stress, lack of motivation, ignorance, lack of training or just being human.

*In case you have a rude nurse, a health worker who is rude that is when it [abuse] can happen* (C13).

There was a mutually reinforcing effect between provider attitude and stress, where stress was said to lead to poor provider attitudes, and their attitudes influenced how they coped with stress.

*Maybe it is the staff’s attitude, if also the staff cannot control her stress, she feels [projects] her stress to the clients* (C31).

#### Provider bias

Implicit and explicit biases appeared to promote favouritism towards certain groups and discrimination against others. Although many providers reported that women are not treated poorly based on any attributes, about 40% acknowledged that there is differential treatment by personal connections, wealth/social status, education, empowerment, age and ethnic affiliations. Providers often contradicted themselves by saying all people are treated the same, but some people are treated differently. Many acknowledged that staff as well as relatives and friends of staff were often given preferential treatment. Preferential treatment to relatives was perceived to be much more of a problem for providers who came from the community they were working in.

*… [preferential treatment] never happens, but if it happens then maybe it is a close relative. Like now we have most of the times our staffs come from around…So those relatives sometimes are treated differently… [I am not from here]. I don’t know them, so I serve then equally, but those who come from around tend to serve their relatives first. They can be given priority or given better treatment* (C26).

Some providers acknowledged a tendency to treat women of higher social status well and to look down on women of low status. Perceptions of wealthier women rewarding providers when they received good treatment were said to promote differential care. Women’s appearances and what they brought with them to the facility also appeared to prompt differential care. As noted, providers were often unhappy when women presented in labour without required items. Since poorer women were more likely to present without these items, this appeared to be a source of differential care. Some providers seemed irritated when women presented to the facility shabbily dressed yet were ‘in love’ with those in nice clothing.

*Some is just physical appearance, you just get in and everybody is in love with her and the other one comes in and everyone is like oooh [laughs] nobody bothers to attend to her, but mostly it is race and financial status* (C4).

*… a person who is well off sometimes when they are coming to deliver and the other mother coming with one cloth…which is torn, they are not treated like the mother who has come with blankets and other things. Sometimes this mother has not bathed there is just that humanity, you just feel that this woman, they don’t treat them equally* (C38).

Providers made judgements based on their initial experiences with women as well as the characteristics of the woman. Providers noted ‘doing the right thing’ even under constraints if they perceived that a woman was well informed or able to advocate for herself.

*It would be different like somebody is from high class or well informed, you will find yourself towards her doing the right thing even when you are straining. Because when I said we have shortage of staff, at times you try to run around but when we know this individual is informed, we will tend to come to that room in most occasions without knowing* (C32).

Women were still expected to be co-operative and respectful: being perceived as empowered or informed was described as resulting in better treatment as long as women did not challenge providers. Providers hinted at stereotypes of who they perceived as well informed or likely to be unco-operative. For example, one provider described teachers as ‘know it alls’, and women from remote areas as more likely to be disrespectful. Institutionalized practices sometimes reinforced individual biases and inadvertently resulted in discrimination towards certain groups. For example, forcing women to stay at the facility against their will because they were unable to pay for services only affected the experiences of poor women, since they were more likely to be unable to pay.

#### Provider training and women’s empowerment

Some providers noted that community perceptions of mistreatment in health facilities were based on past experiences, and providers were now more aware of women’s rights and had changed their behaviours. In particular, they mentioned that verbal and physical abuse was decreasing in prevalence with trainings. Some said they had stopped pinching women since going for training, and they ‘have left the barbaric way of old nursing’.

*I used to do [physically abuse women]. But after I went through a training on the rights of women, I had to change my attitude [Laughs]* (C37).

Lack of knowledge and skills in alternate ways of dealing with difficult situations, as well as unreasonable expectations of the woman in labour appeared to be key reasons for mistreatment. Some providers suggested that training on how to deal with ‘difficult patients’ and on discrimination would enable them to provide better care.

*[training would be useful on] how to handle patients who are hostile and who cannot cooperate…* (C19).

*I think that continuous education will help stop this discrimination issue* (C4).

Providers also thought that training women on their rights and having an accountability mechanism by which women could report mistreatment had reduced disrespect and abuse in some facilities.

*[Abuse of women] used to [happen], but after the training, it stopped. But if it happened, this clients report, as we have the posters with the contact of where to report to in case this happens* (C37).

## Discussion

This article presents data from a mixed-methods study with providers on their perceptions of disrespect and abuse. We use quantitative data to assess their perceptions of the extent of disrespect and abuse and qualitative data to assess drivers of disrespect and abuse. Although most providers reported that women are mostly treated with respect, some acknowledged that verbal and physical abuse, lack of privacy and confidentiality, and discrimination occurs. The drivers of disrespect and abuse included perceptions of women being difficult, stress and burnout, facility culture and lack of accountability, poor facility infrastructure including lack of medicines and supplies, and provider attitudes. Provider bias contributed to discrimination. In addition, provider training and women’s empowerment influenced how women were treated. More than one driver was often at play, with interaction between the different drivers.

The levels of disrespect and abuse reported by providers are higher than that reported by women in other studies in Kenya (Abuya *et al.*, 2015b; Afulani *et al.*, 2019b). For example, in surveys with women in the same county (as part of the larger study), about 11% of women reported some verbal abuse and 4.4% some physical abuse (Afulani *et al.*, 2018b), compared with 53% and 37% of providers reporting seeing verbal and physical abuse, respectively. One potential reason for these differences is the different recall periods: women were reporting on their birth experience in the preceding 9 weeks, whereas providers were not given a defined time period. On the other hand, research based on self-reports from women and observations suggests that women underreport their experiences of disrespect and abuse (Dey *et al.*, 2017; Freedman *et al.*, 2018). Provider perceptions may, therefore, provide an additional perspective on prevalence of disrespect and abuse. Disrespect and abuse must, however, be addressed by centring women’s perceptions and needs—both from a human rights perspective and because negative childbirth experiences reduce utilization of health services (Bohren *et al.*, 2014).

Our findings on drivers of disrespect and abuse are consistent with findings from prior studies. Specifically, disrespect and abuse being justified as needed to save the baby have been previously described (Bohren *et al.*, 2016; Warren *et al.*, 2017). These studies have also highlighted the role of power asymmetry, institutional and health system factors, as well as broader social and gender norms that facilitate disrespect and abuse (Jewkes *et al.*, 1998; Bohren *et al.*, 2016; Warren *et al.*, 2017; Sen *et al.*, 2018). In addition, the role of provider stress and burnout has been acknowledged (Filby *et al.*, 2016; Ndwiga *et al.*, 2017). There has, however, been little discussion of difficult situations and the lack of control or fear of a baby’s death as a traumatic stress that may drive provider behaviour. Similarly, there has been limited discussion in the literature on disrespect and abuse during childbirth on the relationship between providers’ perceptions of women as ‘difficult’, their feeling of helplessness/lack of control, their need to assert power and the resulting abuse of women. Furthermore, the role of provider bias has not been adequately discussed in the work on disrespect and abuse in Africa.

The role of difficult situations in disrespect and abuse emerged as the dominant theme from the qualitative data, although examining this topic was not a goal of the study. Labelling patients as ‘difficult’ is not new in medicine (Klein *et al.*, 1982; Adams and Murray, 1998). It has often been used to describe patients who are medically or interpersonally challenging (Klein *et al.*, 1982; Adams and Murray, 1998). In a study in South Africa, the most ‘undesirable behaviour’ that made nurses to label a patient as ‘bad or difficult’ was that they were unco-operative (Khalil, 2009). Providers often want patients who agree with them and let them be in charge, thus, reward acquiescence when they call patients ‘good’ (Aronson, 2013). Labelling women who do not do what they are told, such as pushing when they are told not to push, is from the expectation that the provider should be in charge, and women should comply. Referring to women as ‘difficult’ implies that providers saw the women as the problem. But provider descriptions of what usually led to abuse were more representative of difficult situations, which were due to a combination of patient characteristics or behaviour, provider characteristics and the environment (Adams and Murray, 1998)—highlighted by the first three themes.

The role of the environment in difficult situations is particularly relevant, given that providers in low-resource settings are chronically exposed to stressors such as high workload; inadequate drugs, supplies and equipment; poor-working conditions; and poor remuneration (Filby *et al.*, 2016; WHO *et al.*, 2016). Prolonged exposure to these stressors without adequate coping mechanisms leads to burnout, which manifests as overwhelming exhaustion, feelings of cynicism, decreased empathy, numbing and reactivity—preventing providers from responding effectively to their patients (Maslach *et al.*, 2001; Bloom, 2010; Tomova *et al.*, 2014). Furthermore, losing a baby is a traumatic experience and stillbirths are frequent in low-resource settings (Blencowe *et al.*, 2016). Trauma theory highlights that traumatic experiences lead to chronic high arousal and exaggerated responses in any situation that may even be remotely connected to the prior trauma (Bloom, 2010). The fear of losing a baby may, therefore, exaggerate provider’s responses in difficult situations, leading to disrespect and abuse. Interventions that enable providers to adequately cope with traumatic and other stressful experiences, as well as address the stressors are thus critical.

In addition, provider narratives suggested frustration, lack of control and helplessness in difficult situations—especially in their use of expressions like ‘I was forced to do it’. It also seemed that when providers felt helpless and anxious, the need to assert power increased and empathy declined, resulting in increased disrespect and abuse. Providers sometimes assert power by rewarding good patients ‘with tender loving care’, and ignoring or delaying care for difficult patients (Khalil, 2009). These approaches to asserting power are, however, not very feasible in the second stage of labour, given the third ‘patient’—the baby whose life providers are invested in saving. The fear of losing a baby also compounds providers’ sense of helplessness and increases their anxiety, which can further suppress empathy and compassion (Tomova *et al.*, 2014; Todd *et al.*, 2015). These factors together may lead them to focus on getting compliance or to react unconsciously. Thus, we conceptualize abuse in this context as a function of provider helplessness, reduced empathy and assertion of power.

The labour and delivery ward in low-resource settings is a prime site for difficult situations: It has ‘patients’ who might be considered difficult because their needs are not being met, providers who have inflexible expectations of how a woman in labour should behave, in combination with stressed and demotivated providers working under challenging conditions. Providers tend to want clarity, order and control; they are satisfied when they have a sense of control over the environment; and frustrated when anything disrupts that order—and when providers feel out of control, tension increases (Adams and Murray, 1998). This may lead to negative emotions such as anger, irritation, fear, hate and hopelessness in difficult situations as demonstrated in our findings. In the unique and dynamic setting of childbirth, it is unlikely that difficult situations can be completely eliminated. Frustration can, however, be minimized by helping providers anticipate the difficulties and prepare for them.

Although most providers denied explicit discrimination, some admitted to biases that led to differential treatment. Differential treatment by social status, ethnicity and connections with provider is supported by research in Kenya and elsewhere (Andersen, 2004; Afulani *et al.*, 2018b, 2019b; Vedam *et al.*, 2019). Difficult situations have also been found to be more likely with patients of lower social class (Crutcher and Bass, 1980). Implicit bias likely plays a role in these disparities. Implicit bias is the unintentional negative or positive evaluation of one group relative to another. It is activated quickly and unknowingly by situational cues such as a person’s skin colour, accent or clothing (Blair *et al.*, 2011; Mendes and Koslov, 2013)—highlighted by provider statements on how their actions were influenced by women’s appearances. Such biases are a reflection of broader societal norms and behaviours (Leape *et al.*, 2012a; Filby *et al.*, 2016). Thus, in societies where gender-based violence, disrespect of the poor, tribalism and differential treatment based on social status are normative, it is not surprising this plays out in the health facility (Andersen, 2004; Filby *et al.*, 2016). Given that providers are higher in the social hierarchy than most women, providers may be more likely to unconsciously treat women of low socio-economic status with disrespect. They are, however, more likely to be conscious of their actions when they meet someone who challenges their social standing, leading to more respectful treatment of women of higher socio-economic status. In addition, research suggests that deeply held biases are more likely to emerge when people are stressed (Mendes and Koslov, 2013). Thus, the high stress of maternity care in under-resourced settings may exacerbate providers’ implicit biases. Programmes that help providers to be more aware of their biases, as well as the implications, and provide them with tools to address these biases may help reduce abuse of the most disadvantaged groups. Recognizing implicit bias is a first step towards minimizing it (Blair *et al.*, 2011). In addition, institutional policies to ensure that individual biases do not influence patient care are needed.

It will take time, motivation, practice and reinforcement to develop provider interpersonal skills and to change their attitudes. Thus, training to prevent disrespect should be part of both pre-service and in-service training. Provider trainings have been shown to increase knowledge of patient rights (Abuya *et al.*, 2015a; Ratcliffe *et al.*, 2016a; Kujawski *et al.*, 2017; Ndwiga *et al.*, 2017). To further decrease disrespect and abuse, trainings should help providers understand factors that lead to difficult situations and to develop alternative ways of dealing with those situations. Trainings should also aim to help providers identify and curb the effects of their biases. Such training should go beyond didactic sessions to more active approaches where providers can practice interpersonal skills in their constrained working conditions and reflect on their values and experiences (Fahey *et al.*, 2013; Abuya *et al.*, 2015a; Afulani *et al.*, 2019a). Beyond training, interventions need to address the factors that contribute to the stressful work conditions such as staff shortages, lack of supplies and medicines, and poor facility infrastructure. Provider disrespect towards women should also be considered in the context of disrespect and power dynamics between different hierarchies of providers to develop a culture of respect in facilities (Leape *et al.*, 2012b). In addition, the role of management and supervision should be considered: providers need to be motivated as well as held accountable for providing dignified and respectful care. Furthermore, interventions beyond the health system are needed, including broader efforts on women’s empowerment and community participation (Abuya *et al.*, 2015a; Ratcliffe *et al.*, 2016a,b; Kujawski *et al.*, 2017).

### Limitations

A key limitation of the study is social desirability bias, as providers might not report poorly about themselves or their facilities. This likely accounts for the much fewer responses on the role of the provider in disrespect and abuse compared with blaming women’s behaviour and the environment. Although providers were less likely to point to themselves as the cause of disrespect and abuse, many acknowledged its occurrence, suggesting social desirability bias may be less of a concern with reporting occurrence. Another limitation is the relatively small sample size; the study was, therefore, not powered for quantitative analysis of potential predictors. Future research with larger samples should examine provider characteristics that may be associated with disrespect and abuse of women. Other limitations include selection bias and generalizability. We used a purposive sample from high-volume facilities. The findings may, therefore, not be representative of all providers in the county. Nonetheless, this is one of the most comprehensive studies in a low-resource setting of provider perspectives on disrespect and abuse involving both clinical and non-clinical providers in different levels of public and private facilities.

## Conclusions

Our findings suggest that disrespect and abuse are driven by difficult situations—real or perceived—in a health system and sociocultural environment that facilitates it. Some women may be difficult for providers to manage, but a woman being difficult alone would probably not lead to abuse without a provider who is stressed, overwhelmed and helpless, but who has power over the woman and works in a culture that tolerates abuse as a means of gaining compliance. Provider bias including implicit biases also influence how women are treated. Interventions, therefore, need to tackle the multiplicity of factors that drive and facilitate disrespect and abuse. To achieve truly dignified and respectful care in health facilities, we need to empower providers with the skills to manage difficult situations, develop positive coping mechanisms for stress, address their biases, as well as change the culture of facilities and health systems. We also need effective incentive systems to motivate and sustain positive behaviour among providers. Pre- and in-service provider training is a first step towards changing provider behaviour and the culture within facilities. This should, however, be part of broader policies to strengthen health systems, create accountability mechanisms and change sociocultural norms.

## Supplementary Material

czaa009_Supplementary_Data
