# Supplementary material for: Providers’ perceptions of disrespect and abuse during childbirth: a mixed-methods study in Kenya
Source: Health Policy Plan. 2020 Mar 10;35(5):577–86. doi: 10.1093/heapol/czaa009 (PMC7225569; doi:10.1093/heapol/czaa009)
Supplement: czaa009_Supplementary_Data [file czaa009_supplementary_data.zip › czaa009_Supplementary_Data/czaa009-Suppl_Data/Table 1.docx]

| **Table 1: Distribution of provider characteristics (N=49)** | | |
| --- | --- | --- |
|  | No. | % |
| Facility type |  |  |
| Government Hospital | 30 | 61.2 |
| Government Health Center | 13 | 26.5 |
| Mission Hospital | 6 | 12.2 |
| Position |  |  |
| Clinical officer/Doctor | 7 | 14.3 |
| Nurse/Midwife | 25 | 51.0 |
| Support staff | 17 | 34.6 |
| Female | 35 | 71.4 |
| Age |  |  |
| <30years | 9 | 18.4 |
| 30-39years | 21 | 42.9 |
| >39years | 19 | 38.8 |
| Married | 39 | 83.0 |
| Years as provider |  |  |
| >6years | 18 | 36.7 |
| 6-10years | 13 | 26.5 |
| >10years | 18 | 36.7 |
| Works more than 5 days a week | 11 | 22.9 |
| Works more than 8 hours per day | 23 | 47.9 |
| From County | 29 | 59.2 |
| <10 years in County | 14 | 28.6 |
|  |  |  |
