# Supplementary material for: Providers’ perceptions of disrespect and abuse during childbirth: a mixed-methods study in Kenya
Source: Health Policy Plan. 2020 Mar 10;35(5):577–86. doi: 10.1093/heapol/czaa009 (PMC7225569; doi:10.1093/heapol/czaa009)
Supplement: czaa009_Supplementary_Data [file czaa009_supplementary_data.zip › czaa009_Supplementary_Data/czaa009-Suppl_Data/Table 2.docx]

|  | | **Table 2: Provider perceptions of extent of disrespect and abuse (N=49)** | | | | |
| --- | --- | --- | --- | --- | --- | --- |
|  |  | | N (%) | | | |
| *Construct* | *Question* | | *No, never* | *Yes, a few times* | *Yes, most of the time* | *Yes, all the time* |
| Respect | Do the doctors, nurses, or other staff at the facility treat women with respect? | | 0(0%) | 4(8.2%) | 26(53.1%) | 19(38.8%) |
| Friendliness | Do the doctors, nurses, and other staff at the facility treat women in a friendly manner? | | 0(0%) | 3(6.1%) | 35(71.4%) | 11(22.4%) |
| Verbal abuse | Do the doctors, nurses, or other health providers shout at, scold, insult, threaten, or talk to women rudely? | | 20(40.8%) | 19(38.8%) | 9(18.4%) | 1(2.0%) |
|  | In your experience at this facility, have you seen this happen? | | 17(34.7%) | 26(53.1%) | 4(12.2%) | 0(0%) |
|  | In your experience at this facility, have you ever done this? | | 23(46.9%) | 22(44.9%) | 4(8.2%) | 0(0%) |
| Physical abuse | Are women treated roughly like pushed, beaten, slapped, pinched, physically restrained, or gagged when they are delivering in the health facility? | | 35(71.4%) | 13(26.5%) | 1(2.0%) | 0(0%) |
|  | In your experience at this facility, have you seen this happen? | | 31(63.3%) | 18(36.7%) | 0(0%) | 0(0%) |
|  | In your experience at this facility, have you ever done this? | | 32(65.3%) | 17(34.7%) | 0(0%) | 0(0%) |
| Privacy and confidentiality | During examinations in the labor room, are women covered up with a cloth or blanket or screened with a curtain so that they do not feel exposed? | | 1(2.0%) | 11(22.4%) | 13(26.5%) | 24(49%) |
|  | Do you think women need privacy during their time in the labor ward? | | 0(0%) | 2(4.2%) | 13(27.1%) | 33(68.8%) |
|  | When women are speaking to the doctors, nurses or other staff at the facility, do you think other people not involved in their care can hear what they are discussing? | | 20(40.8%) | 14(28.6%) | 12(24.5%) | 3(6.1%) |
|  | Do you feel like women’s health information is kept confidential at this facility? | | 1(2.0%) | 2(4.1%) | 23(46.9%) | 22(44.9%) |
| Differential treatment | Will you say women are sometimes treated differently because of their personal attributes, like their age, marital status, number of children, education, wealth, their connections with the facility, or things like that? | | 41(85.4%) | 2(4.2%) | 5(10.4%) | 0(0%) |
|  | Do you think you sometimes treat women differently based on some attributes like their education, wealth, age, marital status, their connections with the facility, or things like that without being aware of it? | | 27(61.4%) | 14(31.8%) | 3(6.8%) | 0(0%) |
| Detention in facility | Are women forced to stay at the health facility against their will because they cannot pay? | | 41(83.7%) | 5(10.2%) | 3(6.1%) | 0(0%) |
